# Role of the VirA histidine autokinase of *Agrobacterium tumefaciens* in the initial steps of pathogenesis

**DOI:** 10.3389/fpls.2014.00195

**Published:** 2014-05-14

**Authors:** Yi-Han Lin, B. Daniel Pierce, Fang Fang, Arlene Wise, Andrew N. Binns, David G. Lynn

**Affiliations:** ^1^Lynn Lab, Department of Chemistry and Biology, Emory UniversityAtlanta, GA, USA; ^2^Binns Lab, Department of Biology, Plant Sciences Institute, University of PennsylvaniaPhiladelphia, PA, USA

**Keywords:** VirA, GAF domain, signal transduction, pathogenesis, two-component system, *Agrobacterium*

## Abstract

Histidine kinases serve as critical environmental sensing modules, and despite their designation as simple two-component modules, their functional roles are remarkably diverse. In *Agrobacterium tumefaciens* pathogenesis, VirA serves with VirG as the initiating sensor/transcriptional activator for inter-kingdom gene transfer and transformation of higher plants. Through responses to three separate signal inputs, low pH, sugars, and phenols*, A. tumefaciens* commits to pathogenesis in virtually all flowering plants. However, how these three signals are integrated to regulate the response and why these signals might be diagnostic for susceptible cells across such a broad host-range remains poorly understood. Using a homology model of the VirA linker region, we provide evidence for coordinated long-range transmission of inputs perceived both outside and inside the cell through the creation of targeted VirA truncations. Further, our evidence is consistent with signal inputs weakening associations between VirA domains to position the active site histidine for phosphate transfer. This mechanism requires long-range regulation of inter-domain stability and the transmission of input signals through a common integrating domain for VirA signal transduction.

## Introduction

Dynamic fluctuations in conformation can be essential for protein function, and large-scale adjustments are often necessary for complex cellular events ranging from allosteric enzymatic activity, regulation of overlapping signal transduction pathways, and the many intra- or inter-subunit protein-protein, protein-DNA, and protein-RNA interactions associated with information flow (Chillemi et al., 2003; Laskowski et al., 2009; Farago et al., 2010). Such protein dynamics are not typically highlighted in static structural models, but can be of critical importance to our understanding of function. The complex roles of the membrane-bound histidine kinases, which function as receptors and signal transducers to modify gene expression or protein function in response to environmental change in many prokaryotes, are critical for committing *Agrobacterium tumefaciens* to pathogenesis (Stock et al., 2000; Mitrophanov and Groisman, 2008; Cheung and Hendrickson, 2010).

**Figure 1:**
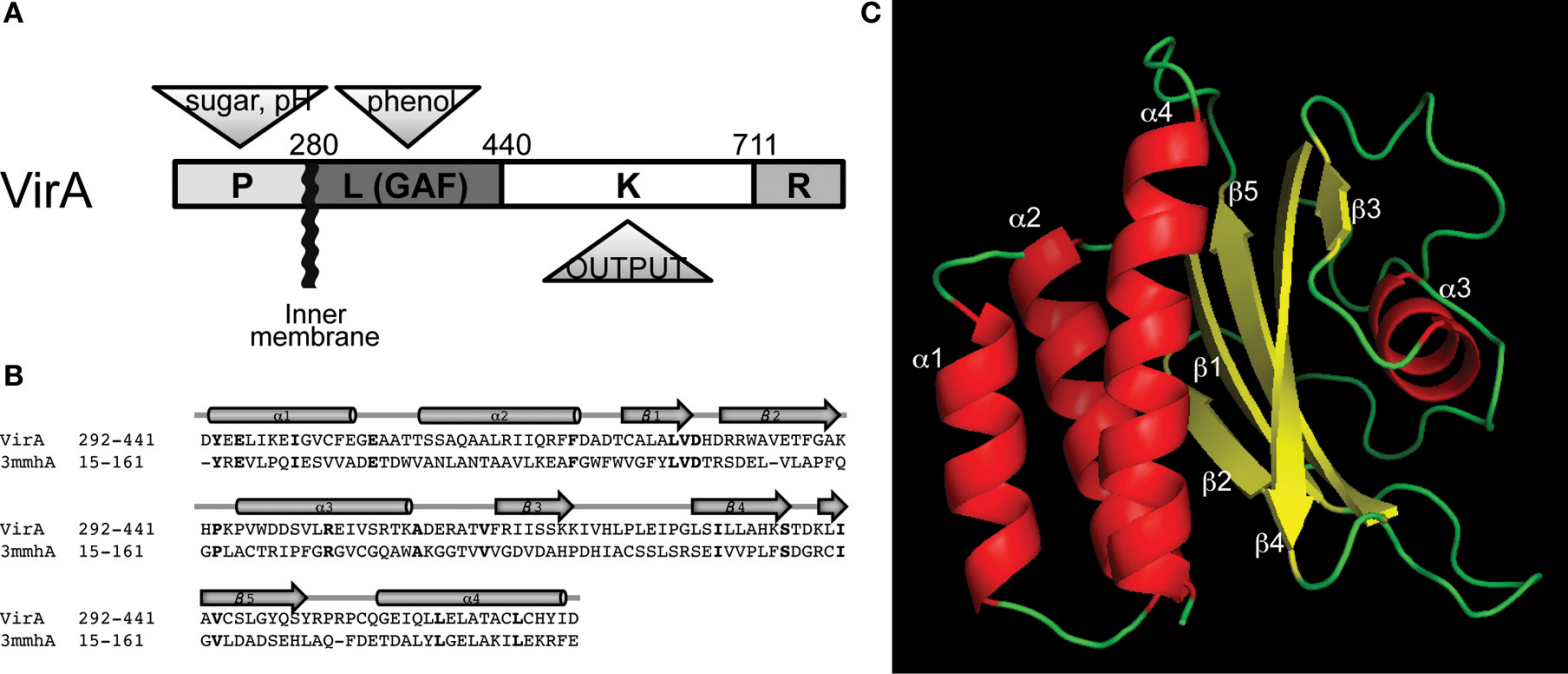
**Domain architecture of the histidine autokinase VirA. (A)** Domain organization and signal inputs of VirA. Besides the conserved kinase (K), three regulatory domains exist to coordinate the signal output. The periplasmic domain (P) perceives sugar and H^+^, the linker domain senses the phenol, and a receiver domain (R) locates at the C-terminus for additional regulation. **(B)** Homology between VirA (292–441) and the *Neisseria meningitidis* fRMsr protein, using Phyre2 (Kelley and Sternberg, 2009). Bold lettering indicates identical residues. **(C)** Predicted structure of the VirA linker region. The GAF-domain containing protein from *N. meningitidis* (PDB ID: 3MMH) provided a template for a predicted protein structure of the VirA linker (292–441).

The VirA histidine kinase and its response regulator VirG form a two-component stimulus-response coupling pair (Gelvin, 2000; Lin et al., 2008). This pair is the necessary first step in the regulation of transcription of the virulence (*vir*) genes on the tumor inducing (Ti) plasmid that ultimately mediate the transfer and integration of DNA into the host cell (Gelvin, 2006; Tzfira and Citovsky, 2006). The multi-domain VirA kinase (Figure 1A) exists as a transmembrane dimer (Pan et al., 1993; Brencic et al., 2004; Wise et al., 2005) and responds to a broad range of phenols (Melchers et al., 1989; Duban et al., 1993) and monosaccharides in low pH environments (Ankenbauer and Nester, 1990; Brencic et al., 2004; Wise et al., 2005; Hu et al., 2013). Maximal expression of the *vir* genes requires a pH sensitive monosaccharide binding to a periplasmic protein ChvE (Ankenbauer and Nester, 1990; Cangelosi et al., 1990). Both ChvE/sugar and phenols associate with VirA to regulate VirG phosphorylation (Chang and Winans, 1992). The terminal receiver domain of VirA homologous to VirG and has been shown to have both negative and positive effects on the phosphorylation cascade (Chang et al., 1996; Wise et al., 2010). Therefore, coordinated actions across the entire VirA dimer appears to be necessary for signal perception and transmission. The central position of the “linker” domain, which joins the trans-membrane helices to the kinase domain, suggests that both periplasmic and cytoplasmic inputs might be integrated here for transmission to the catalytic histidine 474, which is phosphorylated and subsequently used to phosphorylate VirG (Chang and Winans, 1992).

We have used homology models of the VirA linker to gain mechanistic insight for long-range conformational regulation of VirA activity (Wang et al., 2002; Gao and Lynn, 2007). Using mutational and chimeric protein constructs to test prediction, we now document specific interactions within and between VirA domains critical for signal transmission. These long-range structural interactions reveal additional insights into the integrator functions of the linker domain. While it is not yet clear how general these insights may be or why these specific signal inputs have been selected for broad host range evolution, it is certainly clear that sophisticated cooperative motions throughout the entire sensor kinase are exploited for the successful pathogenesis by *Agrobacterium tumefaciens*.

## Materials and methods

### Linker structure modeling

The VirA (292–441) sequence was used to perform a secondary structure homology search using Phyre2 (Kelley and Sternberg, 2009). The GAF domain was common to all but a few of the top 20 hits, and several of these protein structures were known (1VHM, 1F5M) (Gao and Lynn, 2007) (Figure S1A). The top hit was the fRMsr protein from *Neisseria meningitidis*, 12% identity with 93.4% confidence. fRMsr and other hits (e.g., 3P01 and 1F5M) were used as templates for VirA (292–441) (Figure S1B). Comparisons of the resulting GAF domains, including the previous threading of this VirA domain using Swiss Model Workspace (Gao and Lynn, 2007), provided structures that differed only slightly in the relative orientations of the secondary elements (Figure S1C).

### Bacterial strains, plasmids, and reagents

The bacterial strains and plasmids used in this study are listed in Table 1. *E. Coli* strain XL1-Blue (Strategene) was used for routine plasmid construction. Acetosyringone (AS) used for *vir* gene induction was purchased from Sigma-Aldrich Corp. Isopropyl β-d-1-thiogalactopyranoside (IPTG) used to induce protein expression and 5-bromo-4-chloro-3-indolyl-beta-d-galactopyranoside (X-gal) used in library screening were purchased from Research Products International Corp. All cloning reagents were purchased from either New England Biolab or Promega.

**Table 1:** **Bacterial strains and plasmids used in this study**.

**Strains/plasmids**	**Relevant characteristics**	**References**
***E. coli* STRAINS**		
XL1-Blue	*recA1 endA1 gyrA96 thi-1 hsdR17 supE44 relA1 lac*[*F′ proAB lacI^q^Z M15* Tn*10* (Tc^r^)]	Stratagene
***A. tumefaciens* STRAINS**		
A136	Strain C58 cured of pTi plasmid	Watson et al., 1975
A348-3	A136 containing pTiA6NC, Δ*PvirA – virA, virA* deletion, Km^r^	Lee et al., 1992
**PLASMIDS**		
pYW15b	Broad-host-range expression vector, IncW, Ap^r^	Wang et al., 2000
pYW33	*PN25-*6xHis-LZ-*virA(aa285–471)* in pYW15, Ap^r^	Wang et al., 2002
pYW39	*PN25-*6xHis*-virA(aa285–829)(G665D)* in pYW15, Ap^r^	Wang et al., 2000
pYW48	*P_virA_-virA(aa1–829)* in pYW15b, Ap^r^	Wang et al., 2000
pSW209Ω	*virB*::*lacZ*, IncP, Spec^r^	Wang et al., 2000
pJZ4	*P_virB_-lacZ* in pMON596, IncP Spec^r^	Zhang et al., 2000
pJZ6	IncW/ColE expression vector with *P*_*N*25_, Ap^r^	Zhang, Unpublished
pRG109	*P*_*N*25_-His_6_*-virG* in pJZ4, Spec^r^	Gao and Lynn, 2005
pRG150	*lacI^q^* in pJZ4, Spec^r^	Gao and Lynn, 2007
pRG178	*P*_*N*25_-His_6_-LZ(4)-*virA*(*aa426–711*)(*G665D*) in pYW15b, Ap^r^	Gao and Lynn, 2007
pRG179	*P*_*N*25_-His_6_-LZ(3)-*virA*(*aa426–711*)(*G665D*) in pYW15b, Ap^r^	Gao and Lynn, 2007
pRG180	*P*_*N*25_-His_6_-LZ(0)-*virA*(*aa426–711*)(*G665D*) in pYW15b, Ap^r^	Gao and Lynn, 2007
pYL28	*P*_*N*25_-His_6_-*virA*(*aa285–829*)(*C435F*) in pJZ6, Ap^r^	This study
pYL64	*P*_*N*25_*-virA*(*aa438–711*) in pJZ6, Ap^r^	This study
pYL75	*P*_*N*25_*-virA*(*aa285–711*) in pJZ6, Ap^r^	This study
pYL81	*P*_*N*25_*-virA*(*aa446–711*) in pJZ6, Ap^r^	This study
pYL99	*P*_*N*25_*-virA*(*aa426–711*) in pJZ6, Ap^r^	This study
pYL100	*P*_*N*25_*-virA*(*aa460–711*) in pJZ6, Ap^r^	This study
pYL102	*P*_*N*25_*-virA*(*aa453–711*) in pJZ6, Ap^r^	This study
pYL103	*P*_*N*25_*-virA*(*aa467–711*) in pJZ6, Ap^r^	This study
pYL108	*P*_*N*25_*-virA*(*aa426–711*)(*C435F*) in pJZ6, Ap^r^	This study
pYL136	*P*_*N*25_*-virA*(*aa285–829*) in pJZ6, Ap^r^	This study
pYL138	*P*_*N*25_*-virA*(*aa285–829*)(*Q427F*) in pJZ6, Ap^r^	This study
pYL139	*P*_*N*25_*-virA*(*aa285–829*)(*Q427W*) in pJZ6, Ap^r^	This study
pYL140	*P*_*N*25_*-virA*(*aa285–829*)(*C435K*) in pJZ6, Ap^r^	This study
pYL141	*P*_*N*25_*-virA*(*aa285–829*)(*E430K*) in pJZ6, Ap^r^	This study
pYL147	*P*_*N*25_*-virA*(*aa426–711*)(*Q427F*) in pJZ6, Ap^r^	This study
pYL148	*P*_*N*25_*-virA*(*aa426–711*)(*Q427W*) in pJZ6, Ap^r^	This study
pYL149	*P*_*N*25_*-virA*(*aa426–711*)(*E430K*) in pJZ6, Ap^r^	This study
pYL150	*P*_*N*25_*-virA*(*aa426–711*)(*C435K*) in pJZ6, Ap^r^	This study
pYL200	*P*_*N*25_-LZ(4)-*virA*(*aa450–829*) in pJZ6, Ap^r^	This study
pYL201	*P*_*N*25_-LZ(3)-*virA*(*aa450–829*) in pJZ6, Ap^r^	This study
pYL202	*P*_*N*25_-LZ(0)-*virA*(*aa450–829*) in pJZ6, Ap^r^	This study
pYL203	*P*_*N*25_*-virA*(*aa285–829*)(*C435R*) in pJZ6, Ap^r^	This study
pYL205	*P*_*N*25_-LZ(3)-*virA*(*aa450–711*) in pJZ6, Ap^r^	This study
pYL206	*P*_*N*25_-LZ(0)-*virA*(*aa450–711*) in pJZ6, Ap^r^	This study
pYL207	*P*_*N*25_-LZ(4)-*virA*(*aa450–711*) in pJZ6, Ap^r^	This study
pYL212	*P*_*N*25_-*virA*(*aa450–711*) in pJZ6, Ap^r^	This study
pYL213	*P*_*N*25_*-virA*(*aa450–829*) in pJZ6, Ap^r^	This study
pYL214	*P*_*N*25_-LZ(-2)-*virA*(*aa450–829*) in pJZ6, Ap^r^	This study
pYL215	*P*_*N*25_-LZ(-1)-*virA*(*aa450–829*) in pJZ6, Ap^r^	This study
pYL267	*P*_*N*25_-LZ(1)-*virA*(*aa426–711*)(*G665D*) in pJZ6, Ap^r^	This study
pYL268	*P*_*N*25_-LZ(2)-*virA*(*aa426–711*)(*G665D*) in pJZ6, Ap^r^	This study
pYL269	*P*_*N*25_-LZ(-1)-*virA*(*aa426–711*)(*G665D*) in pJZ6, Ap^r^	This study
pYL270	*P*_*N*25_-LZ(-2)-*virA*(*aa426–711*)(*G665D*) in pJZ6, Ap^r^	This study
pYL283	*P*_*N*25_*-virA*(*aa285–711*)(*C449-A-D450*) in pJZ6, Ap^r^	This study
pYL295	*P*_*N*25_*-virA*(*aa285–711*)(*C449-DA-D450*) in pJZ6, Ap^r^	This study
pYL296	*P*_*N*25_*-virA*(*aa285–711*)(*C449-DALK-D450*) in pJZ6, Ap^r^	This study
pYL306	*P*_*N*25_*-virA*(*aa285–711*)(*C449-DAL-D450*) in pJZ6, Ap^r^	This study
pYL307	*P*_*N*25_*-virA*(*aa285–829*)(*K298E*) in pJZ6, Ap^r^	This study
pYL308	*P*_*N*25_*-virA*(*aa285–829*)(*K298E*/*E430K*) in pJZ6, Ap^r^	This study

### Plasmid constructions

**Figure 2:**
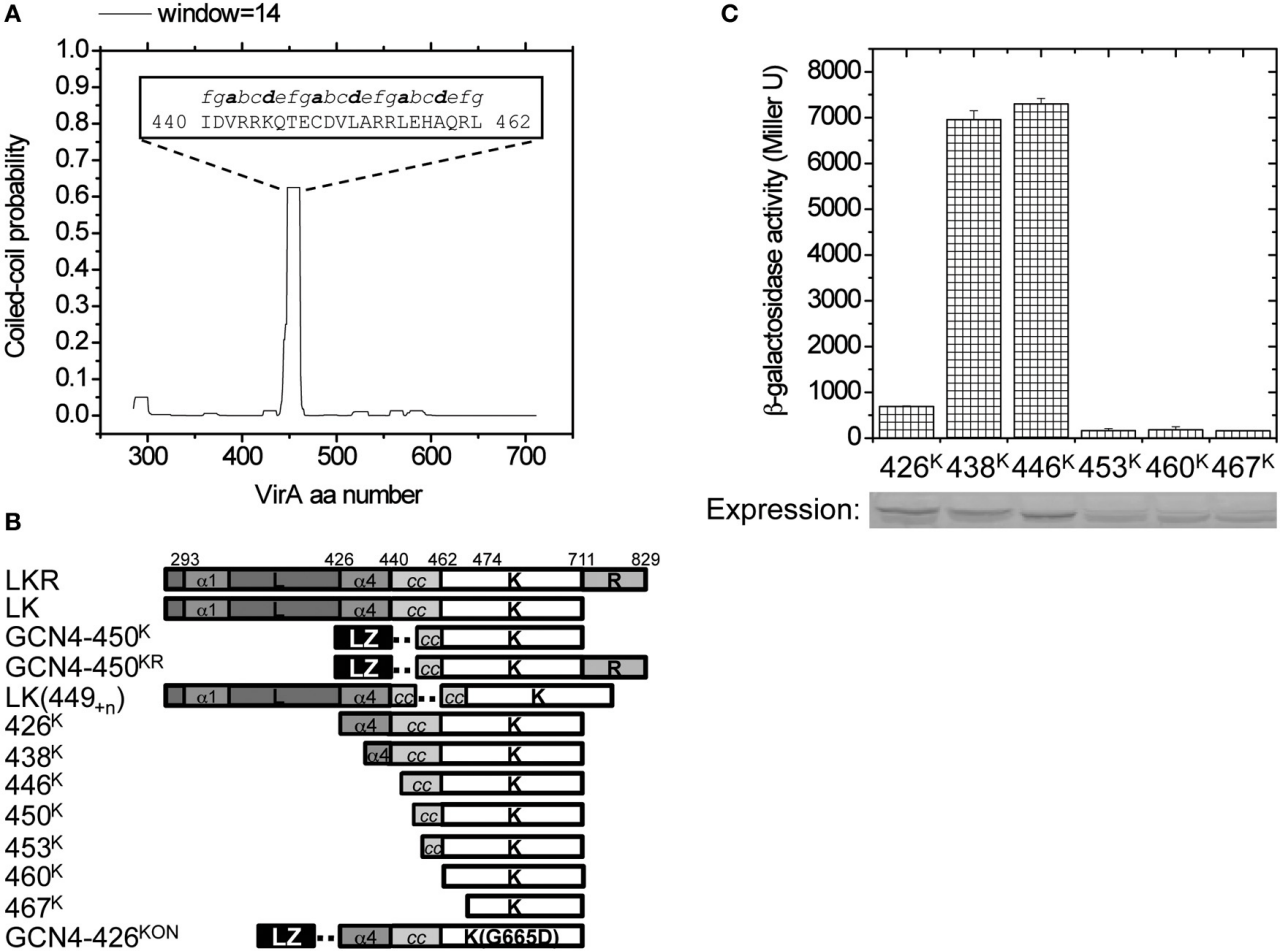
**Design of the VirA variants. (A)** COILS was used to predict coiled-coil forming propensity of VirA-LK(285–711). Aa440–462 was predicted to have high coiled-coil forming probability, and the predicted heptad repeats is shown inside the figure. Heptad positions a and d are shown in bold for orientation. **(B)** The design of VirA truncations, GCN4 fusions, kinase truncations, and direct amino acid insertions. The predicted α1 and α4 of the linker domain and the coiled-coil in K are shown with the dashed line indicating the inserted adapter. **(C)** β-galactosidase activity of different kinase truncations. *A. tumefaciens* strain A136 carrying pRG109 and the kinase truncations from 426^K^ to 467^K^ were assayed for *vir* gene expression in the absence of inducers. *In vivo* protein expression of each truncation was analyzed by Western blot and shown below.

While the scheme for the design of constructs is shown in Figure 2B, plasmid construction procedures are described in Supplementary Materials. The plasmids are listed in Table 1, and the primers are listed in Table S2.

### Library construction and screening

The constitutively active mutants in α4 were identified by randomly mutating aa426–437 in LKR(285–829) via two-step PCR using the primers with an NNN codon replacing each residue, and the results being amplified using primers LKR285 (5′-CGGGATCCGATTGGTTAGCGCGGCGT-3′) and LKRA1 (5′-GCGGTACCGCAACTCTACGTCTTGAT-3′). The library was digested with *BamH*I and *Acc65*I and ligated into the *BamH*I and *Acc65*I digested pJZ6. These constructs were directly transformed into *A. tumefaciens* strain A136 containing pRG109 by eletroporation. To select for the constitutively “on” variants, the transformants of the mutated aa426–437 library were screened on non-inducing media plates containing X-gal. The blue colonies were extracted, sequenced, and the phenotype confirmed by site-directed mutagenesis.

### β-galactosidase assays for vir gene induction

The GCN4 leucine zipper variants, LZ(n)-426^K(G665D)^, were transformed into *A. tumefaciens* strain A348-3 containing pRG150, which has *lacI*^q^ to allow chimera expression only during IPTG induction. The *A. tumefaciens* strains were grown in LB medium with appropriate antibiotics at 28°C to an OD_600_ of 0.4–0.6. The cells were pelleted by centrifugation at 4°C, 7000 × g, for 10 min. The pellet was washed with PBS, and diluted to OD_600_ ~0.1 into tubes containing a total of 1 mL induction medium (Winans et al., 1988) with 200 μM IPTG, and cultured at 28°C, 225 rpm for 15 h. β-galactosidase activity was determined as previously described (Miller, 1972), and the reading of optical densities at 600 and 415 nm was performed using a EL800 microplate reader (BIO-TEK Instruments).

Except for the LZ(n)-426^K(G665D)^ variants, all of the *virA* variants and fusions were transformed into *A. tumefaciens* strain A136 containing pRG109, which carries *P_virB_-lacZ* and *P_N25_-virG*, for *vir* gene expression. The cells were grown and pelleted by the same procedure described above, and diluted to OD_600_ ~ 0.1 into tubes containing a total of 1 mL induction media with or without 300 μM AS, as indicated, and cultured at 28°C, 225 rpm for 15 h. The β-galactosidase activity was determined by the same method as described, from the reading of the optical densities at 600 and 415 nm.

### Immunoblot analysis

*A. tumefaciens* strains were grown in 50 mL LB medium with appropriate antibiotics at 28°C overnight. The cells were harvested by centrifugation at 4°C, 7000 × g, for 10 min. The pelleted cells were washed with PBS and lysed on ice by sonication. The clear lysates were obtained by centrifugation at 4°C, 9000 × g, for 10 min, and analyzed by 10% SDS-PAGE followed by electro-blotting onto nitrocellulose membrane. The membrane was blocked with 3% BSA in TBS, and probed with anti-VirA polyclonal antibody (see SI methods) at 1:200 dilutions. Visualization was achieved using the goat anti-rabbit antibody conjugated with alkaline phosphatase (Amersham) at 1:1000 dilutions, followed by the 1-step NBT/BCIP development (Pierce).

## Results

### Structural model for the linker domain of VirA

The linker domain, designated (L) as it connects TM2 (ending at aa279) with the kinase (K) domain of VirA (Chang and Winans, 1992), was originally defined through mutagenesis and sequence analyses as responsible for phenol signal regulation of kinase activity (Figure 1A). Conservatively selecting residues 292–441 for a Phyre2 secondary structure search revealed 85% of the top 20 hits as GAF domains, so named because of their presence in c*G*MP-regulated cyclic nucleotide phosphodiesterases, *A*denylyl cyclases, and the bacterial transcription factor *F*hlA (Kelley and Sternberg, 2009). Several of these proteins have structural models, and of these, the GAF-domain containing protein fRMsr from *Neisseria meningitis* is the most similar (Gruez et al., 2010). While previous GAF domain-containing proteins are homologous to the VirA linker (Gao and Lynn, 2007), the fRMsr protein (PDB ID: 3MMH) provides a stronger template with 93.4% confidence at 98% coverage, defining the relative positioning of the α-helices and β-sheets (Figure 1B). Using other protein structures as templates gave similar structures with only slight changes in the orientation of the conserved secondary elements (Figure S1).

The resulting threading model of the VirA linker region (Figure 1C) contains a central β-sheet, arranged in a 2-1-5-4-3 strand order (Figure 1C), connected to a helix bundle region composed of α1, α2, and α4 that connects the linker region to the histidine kinase (see Figure 1A). A four-helix bundle architecture, similar to the proposed bundle in VirA, has been characterized in HAMP domains (derived from *H*istidine kinases, *A*denyl cyclases, *M*ethyl-accepting proteins, and *P*hosphatases) (Aravind and Ponting, 1999). These domains regulate signal transmission in histidine kinases (HK) (Falke and Hazelbauer, 2001; Hulko et al., 2006; Airola et al., 2010) and are thought to constitute a dimerization interface (Gao and Lynn, 2007). The α1, α2, and α4 helix region of VirA is proposed to serve as the interface in the VirA dimer based on homologies with these domains.

Initial physical analyses of this model involved over-expressing and purifying the N-terminal His_6_-tagged VirA (285–471) domain (Figure S2A). The relative abundances of secondary structure determined by circular dichroism supported the threading model (~34% α-helix, ~20% β-sheet), but conditions were not found to sufficiently stabilize this truncated domain for further evaluation (Figure S2B). Additional sequence analysis of the full LK domains of VirA (285–711) with COILS (Lupas et al., 1991) identified strong coiled-coil propensity connecting the GAF fold to the N-terminus of the DHp domain, a region in *Thermotoga maritima* HK0853 and *Saccharomyces cerevisiae* Sln1 critical for signal transmission (Tao et al., 2002; Marina et al., 2005). Employing several scanning windows of the heptad repeats, COILS identified aa440–462 (Figure 2A) as having an amphipathic heptad repeat signature. Increasing the size of the scanning window lowered the probability of this region as a coiled-coil, presumably because the sequences surrounding this region do not contribute to the coiled-coil.

To directly evaluate the role of the predicted coiled-coil, we constructed a series of N-terminal truncations of the kinase domain (Figure 2B), starting from amino acid 426 (426^K^), which includes the entire α4 of the linker (L) domain (Gao and Lynn, 2007) and extending through amino acid 467 (467^K^) for complete coiled-coil removal. Most of these truncations appeared stable, but the immunoblot suggests that constructs where the coiled-coil is removed are expressed in lower amounts. To examine how well these VirA fragments are able to induce the *vir* genes, we used a well-characterized β-galactosidase assay where the VirB promoter is placed in front of a plasmid localized *lacZ* gene. As VirA receives the phenol signal, the VirB promoter is turned on and β-galactosidase is produced from the *lacZ* gene. The β-gal activity can then be assayed using its cleavage of the substrate ONPG (Miller, 1972), thereby effectively revealing the activity of the VirA protein. In the absence of inducers, VirA fragments 438^K^ and 446^K^, which retain all or most of the coiled-coil region, have high activity, while partial (453^K^) and complete coiled-coil deletion (460^K^ and 467^K^) are expressed in lower amounts and have a lower activity (Figure 2C). The reduced activity of 426^K^ is striking and consistent with previous evidence that related HAMP-like domains can also be repressive (Gao and Lynn, 2007), suggesting that the 11 amino acids (aa426–437) in α4 contribute to that repression when inducers are absent.

### Functionally connecting the L and K domains

**Figure 3:**
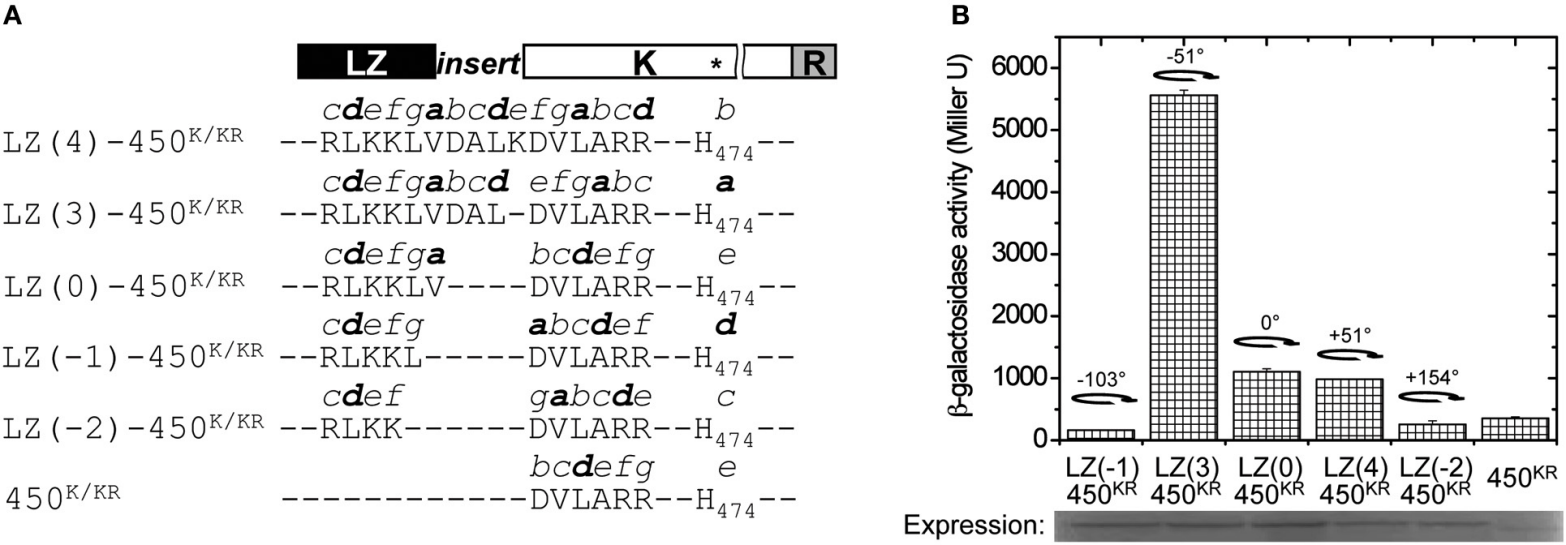
**Chimeric GCN4 fusions with 450^K^ and 450^KR^. (A)** Design of the GCN4-450 fusions. The heptad repeats from *a* to *g* were built from the registry of GCN4 and the adapters. GCN4 enforces the hydrophobic *ad* interface (shown in bold), and shifts the registry of the heptads of kinase coiled-coil according to the different adapters. The predicted position of His474 (^*^in the K domain) in each fusion is shown at the end of the sequence. **(B)** *A. tumefaciens* strain A136 carrying pRG109 and the indicated GCN4-450^KR^ fusions were assayed for *vir* gene expression without inducers. The degree of rotation created by each fusion is shown in the figure with the 0° rotation defined at LZ(0)-450. The protein expression of the GCN4-450^KR^ constructs was analyzed by Western blot.

The helix bundle architecture at the dimerization interface of the GAF-fold in the L domain and the predicted coiled-coil connection to K implies a continuous helical connection being necessary for signal transmission. Previous work describing incremental fusion chimeras with the yeast GCN4 coiled-coil at aa426, just before α4, was interpreted as anchoring the relative position of the helices of the VirA dimer (Wang et al., 2002; Gao and Lynn, 2007). The aa440–462 coiled-coil, however, suggests that in-register fusions with GCN4 are possible, allowing us to define the relative registry of each VirA monomer through to the position of the active site histidine. Fusions were therefore engineered at aa450, removing the N-terminal half of the predicted coiled-coil (Figures 2B, 3A), and placing the fusion just 24 residues upstream of the phosphorylated His474.

While similar results were found using the 450^K^ construct and GCN4 fusions (Figure S3), the effect of helix positioning was more dramatic when the receiver (R) domain is retained in the constructs (Figure 3B). In our experimental conditions, where the constitutive T5 promoter drives VirG expression, the R domain acts as a repressor. The protein expression appeared to be enhanced in all LZ-450^KR^ fusions compared to 450^KR^. The 450^KR^ truncation was active, but the in-register LZ(0)-450^KR^ fusion, which is predicted to place the His474 at the same *e* heptad position, gives a 4-fold increase in activity that may be partially attributed to increased stabilization. A three amino acid insertion, LZ(3)-450^KR^, creating a −51° rotation relative to LZ(0)-450^K^ and moving His474 to the *a* heptad position, shows five times the activity of LZ(0)-450^KR^. A four amino acid insertion, LZ(4)-450^KR^, creating a +51° rotation and positioning His474 at *b*, shows the same level of kinase activity as LZ(0)-450^KR^. The “ON” and “OFF” states being regulated by the relative position of the active site His474 was further tested with LZ(-1)-450^KR^ and LZ(-2)-450^KR^ constructs, corresponding to a rotation of His474 to the *d* and *c* positions on the opposite face of the coiled-coil, and these fusions also showed little activity (Figure 3B).

**Figure 4:**
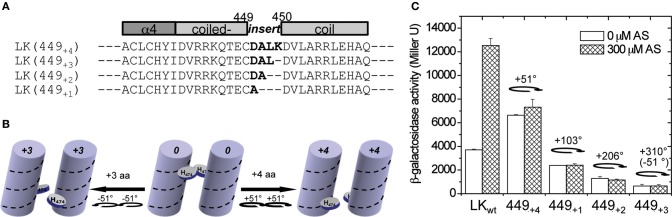
**Direct amino acid insertion within the coiled-coil**. **(A)** The amino acids in bold were inserted between amino acids 449 and 450 in the predicted coiled-coil region. **(B)** An illustration of how His474 moves along the helix coil according to the amino acid insertion at the N′-terminus. **(C)** *A. tumefaciens* strain A136 carrying pRG109 and the LK constructs with different insertions at aa449 (449^+n^) were assayed for *vir* gene expression with or without 300 μM AS. The degree of rotation created by the insertions is shown in the graph.

This model was finally tested by direct insertion of amino acids at residue 449 in the center of the predicted coiled-coil, here denoted as LK(449_+__*n*_) where *n* is the number of amino acids inserted (Figure 4A). As seen in Figure 4B, a +3 amino acid insertion would extend the coiled-coil by almost a single turn and create a −51° rotation, most similar to the LZ(3) fusions, and a +4 amino acid insertion would extend the coiled-coil by more than one turn and create a +51° rotation. The activity observed in LK(449_+1,2,3,4_) constructs follows the general pattern as the GCN4 chimeras in Figure 3, but the predicted registry is different; the +51° rotation LK(449_+4_) enhanced kinase activity while the −51° rotation LK(449_+3_) reduced activity (Figure 4C). We have no direct evidence that the positional variability is due to difference in expression or stability, nor do we know whether the inserts “buckle” or bend the helices in some way to transmit slightly different positional information down the helix to the histidine, and these assignments will require higher structural resolution.

### Mapping the helix association interfaces

**Figure 5:**
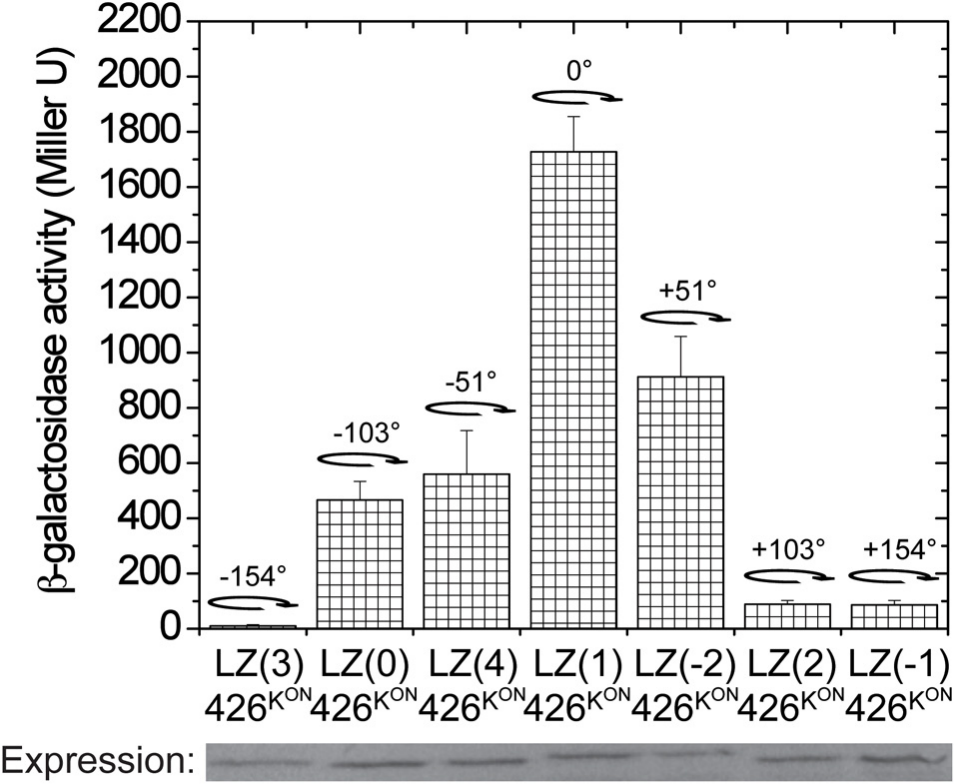
**Signal transmission through α4**. β-galactosidase activity of the GCN4-426^K(G665D)^ fusions. *A. tumefaciens* strain A348-3 carrying pRG150 and the indicated GCN4-426^KON^ fusions were assayed for *vir* gene expression without inducers. 200 μM IPTG was added to induce chimera expression. The degree of rotation created by each fusion is shown in the graph with the 0° rotation defined at LZ(1)-426^KON^.

To further investigate GCN4 fusions for controlling the dimer interface of α4–α4′, the domain was placed outside of the coiled coil region in LK to create GCN4(n)-426^K^ with the same amino acid inserts as in Figure 3A. Since the wild-type 426^K^ has low basal activity (Figure 2C), possibly due to repressive dimer association, a constitutive mutation, G665D, denoted as 426^KON^, was used as before to increase basal activity (Chang et al., 1996; Gao and Lynn, 2007). The full range of GCN4-426^K^ fusions, LZ(0/1/2/-1/-2/3/4)-426^KON^, mapped the possible rotations, and as shown in Figure 5, the activity again follows heptad orientation positioning. The highest activity was found for LZ(1)-426^KON^, and the activity gradually diminished with rotations in either direction. By this analysis, LZ(1)-426^ON^ was assigned as the lowest energy 0 degree rotation interface, and the α4–α4′ dimer interface can be designated as the “ON” conformation (Figure 8A).

The possibility of interactions across α4–α4′ between subunits of the dimer suggests that inputs from sugar/ChvE association might also be transmitted through α1 to the dimer interface in the GAF structure. To test this possibility, we first sought “ON” interface stabilizing mutations within α4 that could provide signal-independent activity. Residues 426–437 of LKR (aa285–829) were randomly mutagenized, and the variants were screened in A136/pRG109 on AB media plates with X-gal without phenolic inducer for active mutants. This approach yielded six constitutive mutants: three with substitutions at Cys435 (C435K, C435R, and C435F), two at Gln427 (Q427W and Q427F), and one at Glu430 (E430K).

**Figure 6:**
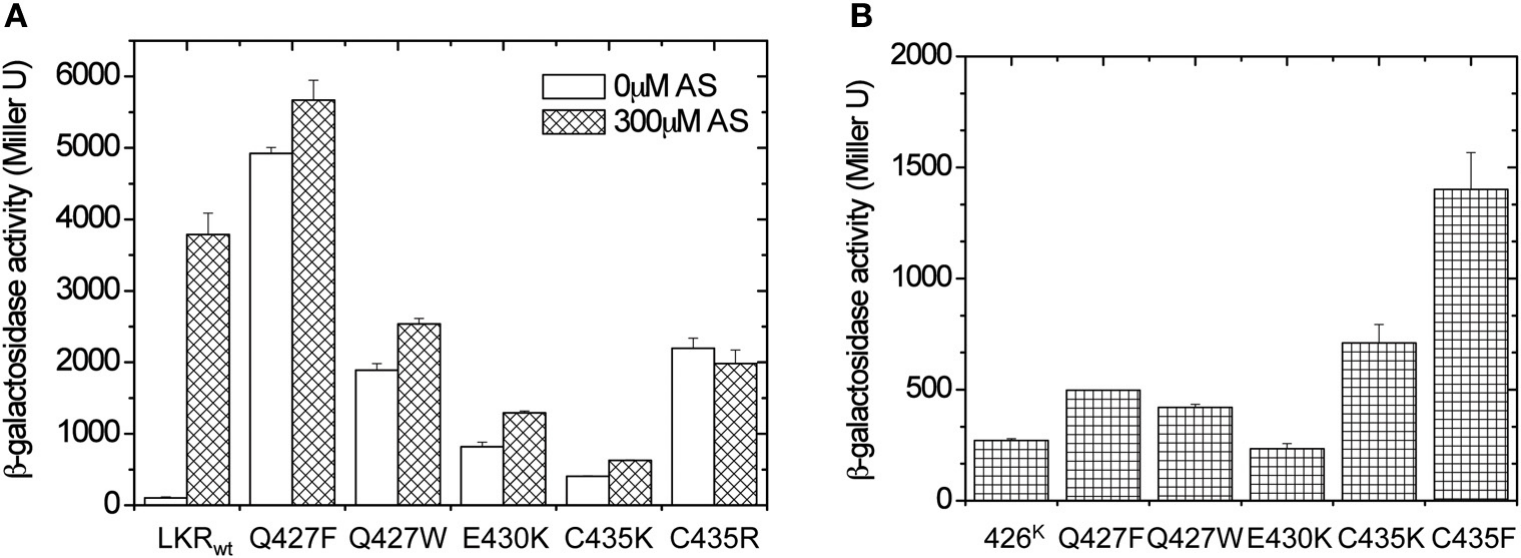
**Library screen for constitutive mutations within α4 (aa426–437). (A)** β-galactosidase activity of the identified constitutively induced mutants. *A. tumefaciens* strain A136 carrying pRG109 and wild-type LKR or LKR mutants were assayed for *vir* gene expression with or without 300 μM AS. **(B)** *A. tumefaciens* strain A136 carrying pRG109 and wild-type 426^K^ or 426^K^ mutants were assayed for *vir* gene expression without inducers.

**Figure 7:**
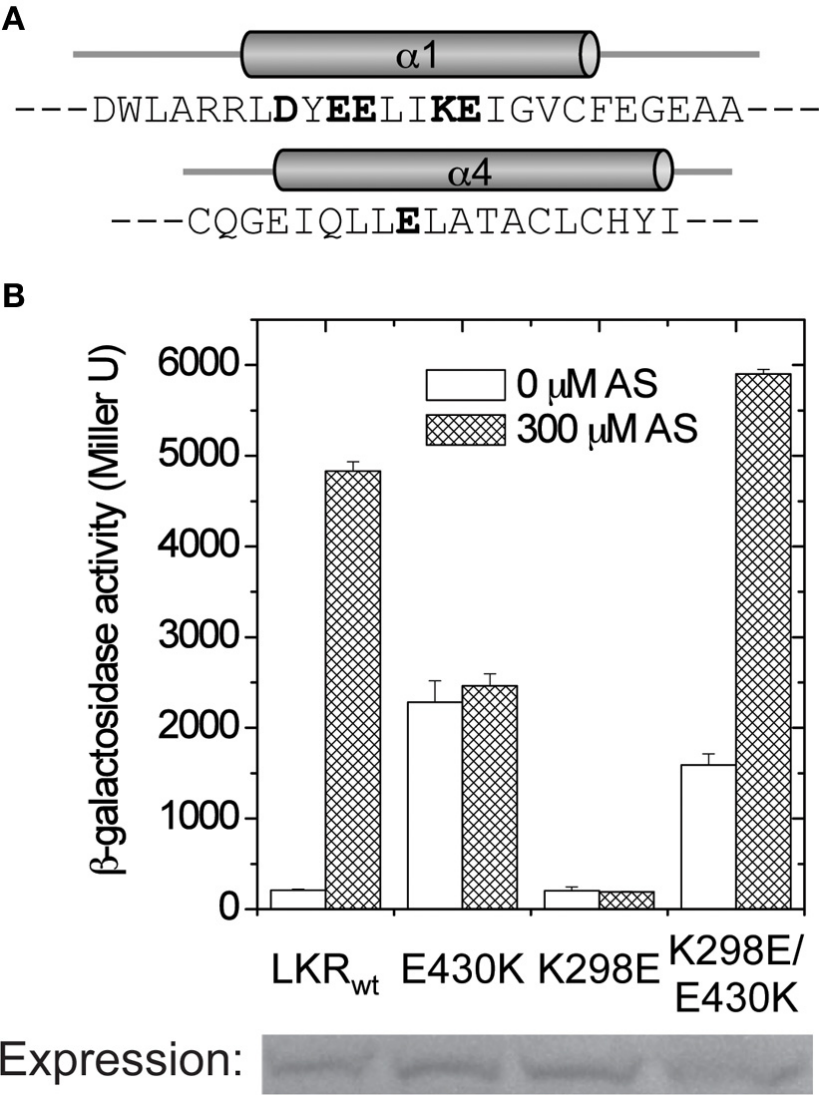
**α1–α4 salt-bridge formation. (A)** The amino acids in linker α1 and α4. The charged residues are shown in bold. **(B)** *A. tumefaciens* strain A136 carrying pRG109 and LKR constructs with E430 and/or K298 mutants were assayed for β-galactosidase activity in the presence or absence of 300 μM AS.

In all of these mutants, phenol induction is severely attenuated (Figure 6A), consistent with the “ON” interface being conformationally stabilized. These mutations were moved to 426^K^ as shown in Figure 6B. The hydrophobic constitutive variants (Q427F, Q427W, C435F) and the charged variant C435K enhanced 426^K^ activity, consistent with stabilization of the “ON” α4–α4′ dimer interface, but the low basal activity of 426^K^(E430K) suggests that its constitutive phenotype in LKR is unlikely a result of α4–α4′ stabilization. The GAF models place several charged residues distributed at the helical surface of α1 (Figure 7A), suggesting that the constitutive phenotype of E430K might result from α4 to α1 charge interaction. While the relative positions of these helices is weakly constrained by these modeling algorithms, among the charged residues in α1, K298 is positioned close enough to form a salt-bridge with E430 in all three models with the allowance of a simple clockwise rotation. To test this possibility, a K298E mutation was constructed to complement E430K. While neither of the single E430K or K298E mutations were phenol responsive, the double mutant (K298E/E430K) restored both kinase activity and phenol inducibility (Figure 7B). This compensating mutation is consistent with an α1 and α4 interface impacting signal transmission, possibly connecting sugar/ChvE binding and phenol induction to conformational transmission through this helical bundle (Gao and Lynn, 2007).

## Discussion

Available protein structures and comparison algorithms have dramatically increased our ability to predict secondary and tertiary folds from primary sequence information. However, determining how these static structures are coupled to function, particularly in proteins not amenable to biophysical and structural analyses, remains a significant challenge. The integral membrane VirA histidine kinase of *Agrobacterium tumefaciens* is an example of remarkable signaling complexity controlling the very first commitments to pathogenesis. We have been able to predict the phenol-sensing linker domain as a GAF fold (Gao and Lynn, 2007), a structure type known to bind cyclic nucleotides, heme, simple chromophores, and branched-chain amino acids (Martinez et al., 2005; Handa et al., 2008), and to regulate secondary messenger metabolism (Sardiwal et al., 2005; Levdikov et al., 2006; Yang et al., 2008). The GAF domain is similar to PAS domains (*P*er-*A*rnt-*S*im), an additional regulatory motif that is involved in protein functional control through interaction with a broad variety of small molecules (Ponting and Aravind, 1997; Hefti et al., 2004). Both GAF and PAS domains are observed in histidine kinases, with an estimated 9 and 33% occurrence, respectively (Gao and Stock, 2009), and successful swaps of those signal sensing domains between different HK have been described (Kumita et al., 2003; Möglich et al., 2009), possibly indicating a common signaling mechanism. The developed structural model has now been used to examine the interactions regulating signal sensing and kinase activation of VirA. Specifically, the helix bundle architecture in the VirA linker and simple rotational motion mediated by these helices was proposed, and this mechanism has been further evaluated with a diverse series of fusions and chimeric constructs.

**Figure 8:**
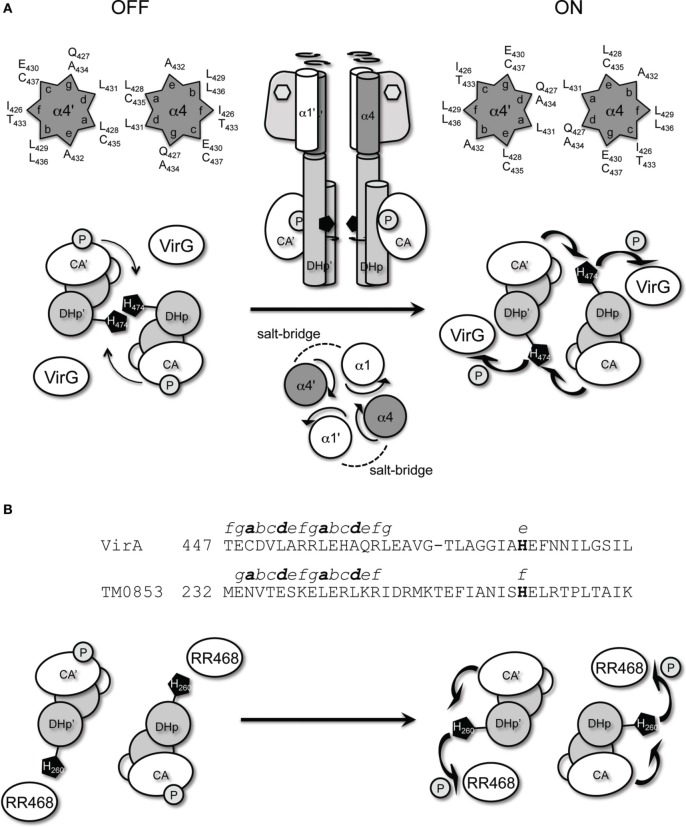
**VirA and *Thermotoga maritima* HK0853**. **(A)** Proposed auto-phosphorylation mechanism of VirA, mediated by α4 coiled-coil. The conserved His474 of VirA, predicted to reside in the dimerization interface, is rotated clockwise upon phenolic sensing to close proximity of the ATP-binding domain at the other subunit for *trans*-phosphorylation and the subsequent VirG phosphoryl transfer. The ON and OFF α4 coiled-coil interface is represented in the helical wheel. **(B)** Sequence alignment of VirA and HK0853 at the coiled-coil region preceding the conserved histidine. The predicted heptads of the coiled-coil of both HK are shown from *a* to *g*, and the conserved histidine are shown in bold. In TM0853, the additional residue in the kinase coiled-coil shifts the registry of the conserved His260 by one residue, which creates a +103° displacement of His260 relative to VirA's His474. Therefore, the same proposed rotation upon signal sensing will move the conserved His260 in TM0853 toward the ATP-binding domain at the same subunit for *cis*-phosphorylation.

The GCN4 leucine zipper motif was used to anchor the orientation of the continuous helix proposed to connect the linker domain and the DHp domain of the kinase. When placed in the middle of the predicted coiled-coil region (aa450), “ON” and “OFF” conformations were identified that could be proposed to arise from different relative orientations of the helices (Figure 8A). Amino acid insertions at the coiled-coil suggested a clockwise rotation mediates VirA activation. The VirA histidine kinase employs a *trans*-phosphorylation mechanism (Brencic et al., 2004), similar to the EnvZ histidine kinase in *E. Coli* (Cai and Inouye, 2003), meaning that the phosphorylation occurs across the subunits of the kinase dimer. A BLAST search identified VirA to have 24% identity to the *Thermotoga maritima* protein HK0853, whose entire cytoplasmic structure has been solved via x-ray crystallography (Marina et al., 2005). If VirA adopts a similar kinase fold as that of HK0853, the predicted clockwise rotation should bring the His474 in VirA closer to the ATP-binding domain of the other subunit for *trans*-phosphorylation (Figure 8A). This model is consistent with previous analyses (Gao and Lynn, 2007), suggesting the rotational motion controls kinase activity at the level of histidine phosphorylation rather than phosphoryl-transfer efficiency. However, HK0853 of *T. maritima* adopts a different *cis*-phosphorylation mechanism (Casino et al., 2009). The difference between VirA and HK0853 can be reconciled by the alignment of the coiled-coil region of both kinases (Figure 8B) and the proposed rotational mechanism. As shown in Figure 8B, the identified coiled-coil region of HK0853 is also located in front of the conserved H-box (Marina et al., 2005). However, when compared with VirA, an additional residue in HK0853 exists between Gly466 and Thr467 of VirA. Having this extra residue in the coiled-coil would shift the conserved histidine of HK0853 (His260) from *e* to *f* in the heptads, which involves a movement of +103° relative to the position of His474 in VirA (Figure 8B). Therefore, the same rotational motion in HK0853 would move His260 from an exposed surface to the ATP-binding domain of the same subunit, requiring a *cis*-phosphorylation mechanism (Figure 8B).

The observation of the high constitutive activities of 438^K^ and 446^K^ is consistent with the argument that the unimpeded kinase is constitutively active while regulatory domains successively repress this activity prior to signal perception release (McCullen and Binns, 2006). The kinase truncation results narrow the repressive region of the linker domain to aa426–437 (Figure 2C), and further lead to the hypothesis that the helical associations within the predicted helical bundle control the critical ON/OFF switch. An “OFF” interface is maintained in the un-induced state, and signal sensing switches it to the “ON” interface. Successful engineering of rotational motions at this region by similar GCN4 fusions displayed a clear rotational activation (Figure 5), and predicts the ON/OFF interface of α4–α4′ (Figure 8A). Furthermore, the control by GCN4 at both 426^K^ and 450^K^ indicates the rotational motion is coherently transmitted from the linker domain to the kinase core. Indeed, library screens for constitutive mutants identified both hydrophobic and electrostatic interactions stabilizing the dimerization interface at α4–α4′. A recent study on an engineered HK YF1 (generated by replacing the oxygen-sensing PAS domain of *Bradyrhizobium japonicum* FixL with the FMN (flavin-mononucleotide)-binding LOV (light-oxygen-voltage) domain from *Bacillus subtilis* YtvA) provided structural insight into the coiled-coil motifs mediating signal transmission between functional domains (Diensthuber et al., 2013). Furthermore, it also implies a simple motion and a fundamental mechanism that can be shared between different signal sensing domains for kinase output.

And most interestingly, this search for functional long-range interactions identified the α1 helix as a key regulator for signal activation in this rotational mechanism. Salt-bridge associations between K298 (α1) and E430 (α4) is consistent with the computational model of the helix bundle containing α1 and α4 interfaces in the VirA dimer (Wang et al., 2002) and its regulator role in signal transmission. The other charged residues in α1 were previously found to be important in controlling a “piston-like” motion, mediated by the monosaccharide/H^+^ sensing from the periplasmic domain (Gao and Lynn, 2007). Therefore, this bundle may be the conversion point for both sugar/pH and phenol inputs to counteract the repressive region in the dimerization interface at α4–α4′. In addition, preliminary chemical cross-linking results aimed at clarifying the receiver domain's role in enhancing signal response precision indicated an association with the kinase core at this coiled-coil region (Figure S4), but the nature of this association is not yet clear.

The identified interactions point to highly cooperative long-range motions transmitting signal association within the VirA dimer to regulate the very first steps of pathogenesis. The positioning of α1, α2, and α4 vary in the three structural models for the GAF domain and indeed these kinds of structural details are the least well-defined in the structural algorithms. Figure 8 outlines a mechanistic model that is consistent with our chimeric fusion, but the nature of the long-range transmission (Gao and Stock, 2009) has also implicated symmetry switching models (Moore and Hendrickson, 2012). A recent structural analysis identified a critical proline residue in CpxA that contributes to helix bending in that kinase (Mechaly et al., 2014), but that residue is not conserved in VirA. The range of constructs prepared here provide opportunities to identify constructs amenable to direct structural analyses and further evaluation of these models. Most importantly, the remarkably coordinated action of VirA in processing three separate input signals likely contributed significantly to the success of this pathogen. These constructs now allow the system to be simplified sufficiently to define which signal is being processed and to map the signaling landscape of the host wound site for commitment to pathogenesis.

### Conflict of interest statement

The authors declare that the research was conducted in the absence of any commercial or financial relationships that could be construed as a potential conflict of interest.
