# Supplementary material for: Role of the VirA histidine autokinase of Agrobacterium tumefaciens in the initial steps of pathogenesis
Source: Front Plant Sci. 2014 May 14;5:195. doi: 10.3389/fpls.2014.00195 (PMC4030172; doi:10.3389/fpls.2014.00195)
Supplement: Supplementary file 1 [file DataSheet1.DOCX]

***Supplementary Material***

**Role of the VirA histidine autokinase of *Agrobacterium tumefaciens* in the initial steps of pathogenesis**

**Authors and Affiliations:**

*Yi-Han Lin‡, Fang Fang‡, B Daniel Pierce‡, Arlene Wise°, Andrew N Binns°, and David G Lynn‡*

*‡Departments of Chemistry and Biology, Emory University, Atlanta, GA 30322*

*°Plant Sciences Institute, Department of Biology, University of Pennsylvania, Philadelphia, PA 19104*

**Supplementary Materials:**

*Plasmid construction.* The *Agrobacterium* expression vector used throughout this study is pJZ6, which drives the protein expression by the coliphage T5 *P*_N25_ promoter. All of the *virA* variants or GCN4 fusions were introduced into pJZ6 with *BamH*I and *Acc65*I sites. The GCN4 leucine zipper fusions LZ(n)-*virA(450-829)* were created by the two-step PCR method, in which the GCN4 portion was amplified from pYW33 with YL95 and the corresponding reverse primers, and the *virA* portion was amplified from pYW48 with the corresponding complementary forward primers and LKRA1. The amplified DNA was digested with *BamH*I and *Acc65*I, and introduced into pJZ6 to generate pYL200, pYL201, pYL202, pYL214, and pYL215. pYL201, pYL202, and pYL200 were served as the templates for amplification with YL95 and LKKpnI, followed by *BamH*I and *Acc65*I digestion and ligation with pJZ6, to generate pYL205, pYL206, and pYL207. LZ(n)-*virA(426-711)(G665D)* fusions were also generated by the same two-step PCR method, except using pYW39 as the template for *virA(G665D)*, and resulted into pYL267, pYL268, pYL269, and pYL270.

The coiled-coil amino acid insertion of LK was also generated by the two-step PCR method with LKR285, LKKpnI, and the corresponding complementary primers. The resulted LK(449_+n_) constructs were pYL283, pYL295, pYL296, and pYL306. The *virA* truncations were generated by PCR from pYW48 with corresponding forward primers and the reverse primers LKKpnI or LKRA1, and introduced into pJZ6 to create pYL64, pYL75 (LK), pYL81, pYL99, pYL100, pYL102, pYL103, pYL136 (LKR), pYL212, and pYL213.

The LKR mutants were generated by the two-step PCR from pYW48 with LKR285, LKRA1, and the corresponding complementary primers to introduce the mutations. Followed by *BamH*I and *Acc65*I digestion, the DNA fragment was introduced into pJZ6 to generate pYL138, pYL139, pYL140, pYL141, pYL203, and pYL307. 426^K^(C435F) and 426^K^(C435K) were generated by PCR using pYL28 and pYL140 as the templates and YL134 and LKKpnI as the primers. After *BamH*I and *Acc65*I digestion and the successive ligation into pJZ6, pYL108 and pYL150 was created. The other 426^K^ variants with constitutive mutations were generated by the one-step PCR from their LKR constructs pYL138, pYL139, and pYL141, with primers YL214, YL215, YL216, and LKKpnI, and resulted into pYL147, pYL148, and pYL149. LKR(K298E/E430K) was generated by the two-step PCR using pYL141 as the template and the corresponding complementary primers to introduce K2983E resulted into pYL308.

N-terminal His_6_-tagged receiver domain (*virA712-829*) was generated by PCR from pYW48 with primers YL5 and YL6, followed by *Sac*I and *Kpn*I digestion, and ligated into *Sac*I and *Kpn*I digested pYW15b to generate pYL3. His_6_-tagged linker (*virA285-444*) was generated by PCR from pYW48 with primers LKR285 and YL103, followed by *BamH*I and *Acc65*I digestion and ligated into *BamH*I and *Acc65*I digested pQE30 to generate pYL69. The nucleotide sequence of all the plasmid constructs were confirmed by DNA sequencing facility performed by Agencourt Genomic Service (now Beckman Coulter Genomics).

*Protein expression and purification.* *E. coli* M15[pREP4] strain carrying the indicated linker or receiver constructs were grown in LB medium at 37 ^o^C, 200 rpm overnight. The overnight culture was diluted 1:100 into fresh LB medium, and subcultured at 37 ^o^C, 200 rpm to an OD_600_ of 0.6-0.7. The cell culture was cooled down to 20 ^o^C, and 0.05 mM isopropyl-β-D-thiogalactopyranoside (IPTG) was added to induce protein overexpression. Cells were grown at 20 ^o^C, 200 rpm, for an additional 14-18 hours, and harvested by centrifugation at 4 ^o^C, 6000 rpm, for 10 min. The cell pellet was washed with 0.9% NaCl, re-suspended in lysis buffer, and lysed on ice by sonication (ON pulse: 1 sec; OFF pulse: 2 sec; total pulse: 2 min). The clear lysate was obtained by centrifugation at 4 ^o^C, 9000 x g, for 30 min. The His_6_-tagged protein was purified with Ni-NTA affinity column following the protocol in The Qia*expressionist* (Qiagen). The purified protein was exchanged into PBS buffer, pH 6.4, by the 10K Amicon Ultra (Millipore). All the buffers for purification and protein storage contain an additional 10% glycerol to enhance protein stability.

*Circular Dichroism.* 40 μL purified protein was placed into a quartz cuvette with 0.1 mm pathlength. Each spectrum was obtained by the average of three successive scanning from 260 nm to 190 nm at a scanning rate of 100 nm/min with a resolution of 0.2 nm, using Jasco-J180 spectropolarimeter. The ellipticity [θ]_obs_ was converted to the mean-residue-ellipticity ([θ], deg cm^2^ dmol^-1^) by the equation [θ] = ([θ]_obs_×100×*Mr*) / (*c×l×N_A_*), in which *Mr* is the molecular weight of the protein in Dalton, *c* is the protein concentration in mg/mL, *l* is the pathlength of the cuvette in cm, and *N_A_* is the number of the residues of the protein.

*Chemical cross-link.* The purified linker and receiver domains were cross-linked with dimethyl suberimidate (DMS) at the ratio of 1:20 and 1:50 (m/v). The reaction was performed at room temperature for 1 hour before quenched by the addition of SDS-PAGE sample buffer. The samples were boiled for 10 minutes, followed by 15% SDS-PAGE analysis and electro-blotting onto nitrocellulose membrane. The membrane was blocked with 3% BSA in TBS, and probed with the monoclonal anti-polyhistidine antibody (Sigma). Visualization was achieved using the goat anti-mouse antibody conjugated with alkaline phosphatase (Pierce) at 1:1000 dilutions, followed by the 1-step NBT/BCIP development (Pierce).

**Supplementary Figures:**

A

| **PDB ID** | **Chain** | **Protein** | **Confidence** | **% Identity** |
| --- | --- | --- | --- | --- |
| 3MMH | A | methionine-r-sulfoxide reductase | 95.6 | 13 |
| 4G3V | B | nlh2 gaf domain (inactive) | 93.6 | 11 |
| 3P01 | C | two-component regulator | 88.6 | 11 |
| 4BWI | A | phytochrome cph2 | 88.0 | 14 |
| 3HCY | B | two-component sensor 2 histidine kinase | 86.7 | 13 |
| 3E0Y | A | unknown | 86.5 | 12 |
| 3TRC | A | phosphoenolpyruvate-protein phosphotransferase | 85.5 | 10 |
| 3P01 | A | two-component regulator | 84.2 | 9 |
| 4G3K | B | transcriptional regulator nlh1 | 80.3 | 11 |
| 3CI6 | B | phosphoenolpyruvate-protein phosphotransferase | 79.3 | 12 |
| 2VJW | A | hydrolase | 77.1 | 12 |
| 3EEA | B | unknown | 74.6 | 11 |
| 1VHM | A | GAF domain-like | 74.1 | 13 |
| 1MC0 | A2 | GAF domain-like | 73.1 | 12 |
| 2QYB | A | membrane protein | 72.4 | 11 |
| 3K2N | B | transcriptional regulator sigma-54-dependent | 66.8 | 16 |
| 3RFB | B | oxidoreductase | 65.2 | 13 |
| 3OOV | A | methyl-accepting chemotaxis protein | 57.2 | 8 |
| 2W3G | A | two-component histidine kinase | 54.1 | 12 |
| 2VEA | A1 | GAF domain-like | 52.2 | 11 |

B C


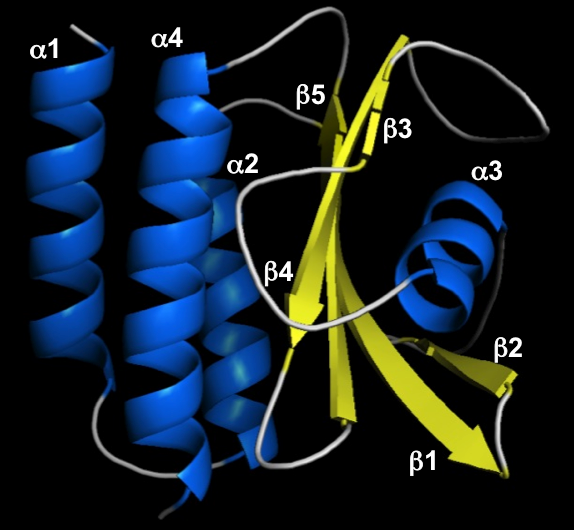

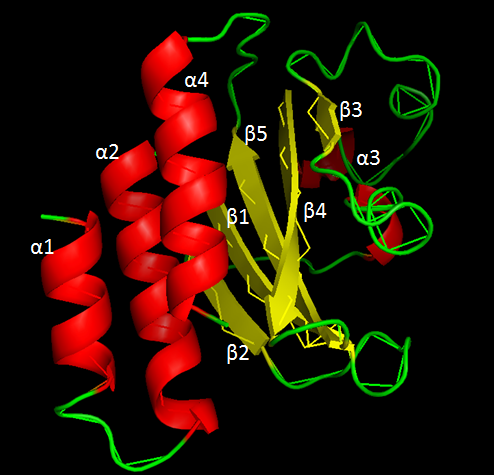


**Supplementary Figure 1. Modeling of the histidine autokinase VirA. A)** Phyre2 results using VirA (292-441) (28) showing 85% of the top 20 hits as GAF domains. Confidence is given on a scale of 1-100, calculated using the algorithm provided by Phyre2. **B)** Structural model of the VirA linker, developed using Phyre2 *Nostoc sp. PCC7120* two-component response regulator protein (PDB ID: 3P01) as the template. **(C)** Predicted structure of the VirA linker region using Swiss Model Workspace with yeast protein YKG9 as the template (PDB ID: 1F5M).

A B

**Supplementary Figure 2. *In vitro* characterization of the linker domain. A)** Purification of the linker domain. N-terminus His_6_-tagged VirA(285-471) was over-expressed by 0.2 mM IPTG induction for 18 hours at 16 ºC. The protein was purified with Ni-NTA affinity resin (Qiagen). Lane 1: whole cell; lane 2: soluble lysate; lane 3: flow-through of Ni-NTA; lane 4: wash-off; lane 5-9: eluted fractions. **B)** Circular dichroism spectrum of 15 μM purified linker and the calculated % secondary structure. The % secondary structure of the linker was calculated by three programs: CONTILL, CDSSTR, and SELCON3. The secondary structure of the GAF domain 1F5M is also listed.


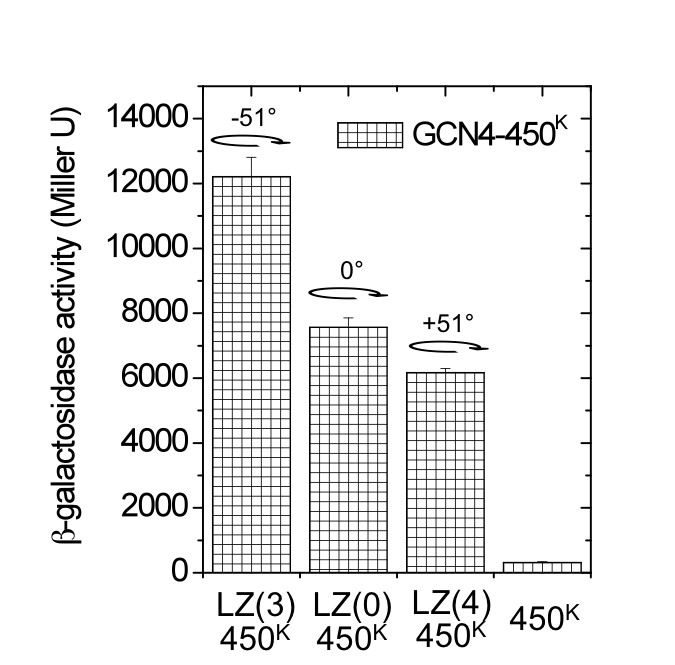


**Supplementary Figure 3.** **Chimeric GCN4 fusions with 450^K^.** *A. tumefaciens* strain A136 carrying pRG109 and the indicated GCN4-450^K^ fusions were assayed for *vir* gene expression without inducers. The degree of rotation created by each fusion is shown in the figure with the 0 degree rotation defined at LZ(0)-450.

A


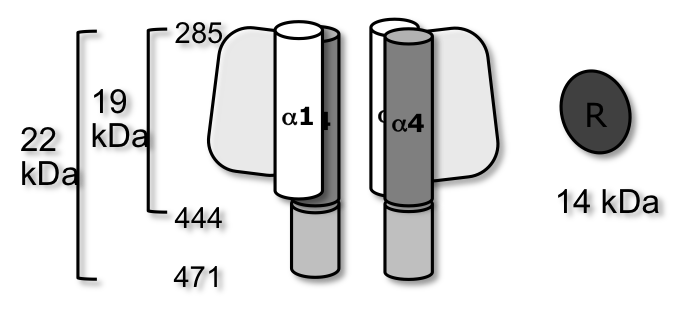


B


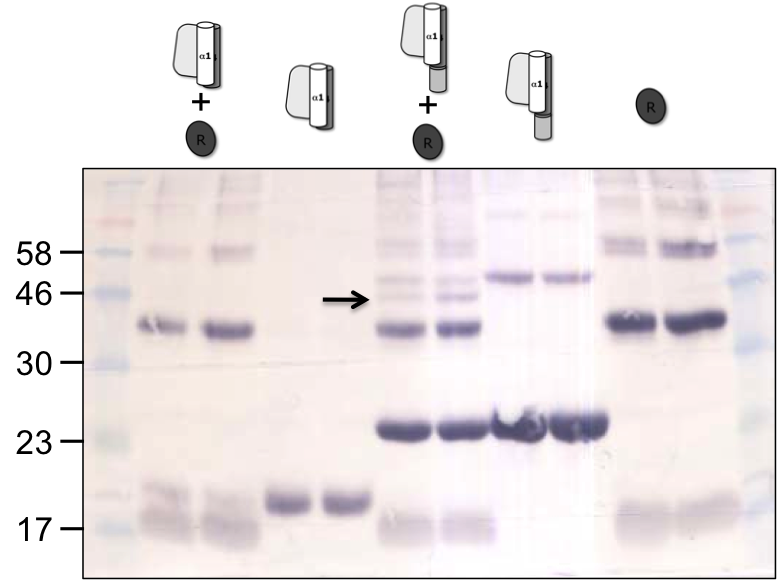

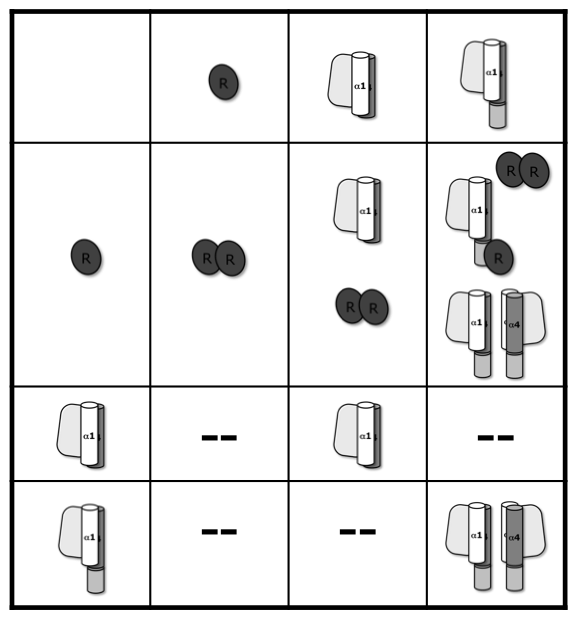


**Supplementary Figure 4.** **Chemical cross-link of the VirA linker and receiver domains. A)** Schematic representation of the two linker constructs (aa285-444; aa285-471), the receiver domain (aa712-829), and their corresponding molecular weight. The chemical structure of the cross-linker DMS is also shown. **B)** Western blotting by anti-His_6_ antibody to probe both the linker and the receiver was used to detect the results of chemical cross-linking. Two molar ratios of protein:DMS (1:20, 1:50) were used, and shown in parallel. Lane 1, 2: linker(285-444)+R; lane 3, 4: linker(285-444) alone; lane 5, 6: linker(285-471)+R; lane 7, 8: linker(285-471) alone; lane 9, 10: R alone. The cross-linked heterodimer of linker(285-471) and receiver is marked with an arrow. The schematic representation of the cross-linking result is shown in the table.

**Supplementary Tables:**

**Supplementary Table 1:** **Plasmids used in supplementary materials**

| Strains/plasmids | Relevant characteristics | Reference | |
| --- | --- | --- | --- |
| pRG15 | *P*_N25_-His_6_-*virA*(*aa285-471*) in pBS, Ap^r^ | | ([Wang, Gao et al. 2002](#_ENREF_138)) |
| pYL3 | *P*_N25_*-virA*(*aa712-829*) in pYW15b, Ap^r^ | | This chapter |
| pYL69 | *P*_N25_*-virA*(*aa285-444*) in pQE30, Ap^r^ | | This chapter |

**Supplementary Table 2:** **Primers used in this study**

| Primer | Characteristic | Sequence (5’🡪3’) (RE site; *mutation*, **insertion**) |
| --- | --- | --- |
| LKR285 | BamHI_285_for | CG GGA TCC GAT TGG TTA GCG CGG CGT |
| LKRA1 | KpnI_stop_829_rev | GC GGT ACC GCA ACT CTA CGT CTT GAT |
| LKKpnI | KpnI_stop_711_rev | GG GGT ACC CTA ACG CGG TGC CTT ATT GCG |
| YL5 | SacI_712_for | GCT GAG CTC GGA AAC GGG GAG ATT GTG GC |
| YL6 | KpnI_stop_829_rev | GCT GGT ACC TGA CAC GTC GCA ACT CTA CGT C |
| YL95 | BamHI_LZ_for | GG GGA TCC GGA GGT TGC GGA GGT AAG |
| YL50 | C435F_for | C ACC GCC *TTC* CTC TGT CAC |
| YL51 | C435F_rev | GTG ACA GAG *GAA* GGC GGT G |
| YL103 | KpnI_stop_444_rev | CC GGT ACC TCA ACG CCG AAC ATC GAT ATA G |
| YL106 | BamHI_438_for | CC GGA TCC CAC TAT ATC GAT GTT CGG |
| YL110 | BamHI_446_for | GG GGA TCC CAG ACC GAA TGC GAC GTT TTG G |
| YL134 | BamHI_426_for | GG GGA TCC ATT CAG CTT CTT GAA CTC G |
| YL135 | BamHI_453_for | GG GGA TCC GCC AGA CGA TTG GAG CAT G |
| YL136 | BamHI_460_for | GG GGA TCC CAA CGC CTT GAG GCA GTT G |
| YL137 | BamHI_467_for | GG GGA TCC ACA CTT GCC GGC GGA ATA G |
| YL165 | L429X_for | CAG CTT *NNN* GAA CTC GCC ACC |
| YL166 | L429X_rev | GGT GGC GAG TTC *NNN* AAG CTG |
| YL167 | E430X_for | CAG CTT CTT *NNN* CTC GCC ACC |
| YL168 | E430X_rev | GGT GGC GAG *NNN* AAG AAG CTG |
| YL169 | L431X_for | CTT CTT GAA *NNN* GCC ACC GCC |
| YL170 | L431X_rev | GGC GGT GGC *NNN* TTC AAG AAG |
| YL171 | A432X_for | CTT GAA CTC *NNN* ACC GCC TGC |
| YL172 | A432X_rev | GCA GGC GGT *NNN* GAG TTC AAG |
| YL175 | A434X_for | CTC GCC ACC *NNN* TGC CTC TGT C |
| YL176 | A434X_rev | G ACA GAG GCA *NNN* GGT GGC GAG |
| YL177 | L436X_for | CC ACC GCC TGC *NNN* TGT CAC |
| YL178 | L436X_rev | GTG ACA *NNN* GCA GGC GGT GG |
| YL179 | C437X_for | GCC TGC CTC *NNN* CAC TAT ATC G |
| YL180 | C437X_rev | C GAT ATA GTG *NNN* GAG GCA GGC |
| YL181 | I426X_for | CGA CCT TGC CAA GGC GAA *NNN* CAG CTT CTT GAA CTC GCC |
| YL182 | I426X_rev | GGC GAG TTC AAG AAG CTG *NNN* TTC GCC TTG GCA AGG TCG |
| YL183 | Q427X_for | CCT TGC CAA GGC GAA ATT *NNN* CTT CTT GAA CTC GCC ACC |
| YL184 | Q427X_rev | GGT GGC GAG TTC AAG AAG *NNN* AAT TTC GCC TTG GCA AGG |
| YL185 | L428X_for | TGC CAA GGC GAA ATT CAG *NNN* CTT GAA CTC GCC ACC GCC |
| YL186 | L428X_rev | GGC GGT GGC GAG TTC AAG *NNN* CTG AAT TTC GCC TTG GCA |
| YL187 | T433X_for | CAG CTT CTT GAA CTC GCC *NNN* GCC TGC CTC TGT CAC TAT |
| YL188 | T433X_rev | ATA GTG ACA GAG GCA GGC *NNN* GGC GAG TTC AAG AAG CTG |
| YL189 | C435X_for | CTT GAA CTC GCC ACC GCC *NNN* CTC TGT CAC TAT ATC GAT |
| YL190 | C435X_rev | ATC GAT ATA GTG ACA GAG *NNN* GGC GGT GGC GAG TTC AAG |
| YL191 | Q427F_for | TGC CAA GGC GAA ATT *TTC* CTT CTT GAA CTC GCC |
| YL192 | Q427F_rev | GGC GAG TTC AAG AAG *GAA* AAT TTC GCC TTG GCA |
| YL193 | Q427W_for | CAA GGC GAA ATT *TGG* CTT CTT GAA CTC |
| YL194 | Q427W_rev | GAG TTC AAG AAG *CCA* AAT TTC GCC TTG |
| YL197 | C435K_for | GAA CTC GCC ACC GCC *AAA* CTC TGT CAC TAT ATC |
| YL198 | C435K_rev | GAT ATA GTG ACA GAG *TTT* GGC GGT GGC GAG TTC |
| YL199 | E430K_for | CAG CTT CTT *AAA* CTC GCC ACC |
| YL200 | E430K_rev | GGT GGC GAG *TTT* AAG AAG CTG |
| YL214 | BamHI_426_Q427F | GG GGA TCC ATT *TTC* CTT CTT GAA CTC GCC ACC |
| YL215 | BamHI_426_Q427W | GG GGA TCC ATT *TGG* CTT CTT GAA CTC GCC ACC |
| YL216 | BamHI_426_E430K | GG GGA TCC ATT CAG CTT CTT *AAA* CTC GCC ACC |
| YL250 | LZ(4)-450^K^_ for | G AAG AAG CTG GTC **GAC GCA CTG AAG** GAC GTT TTG GCC AGA |
| YL251 | LZ(4)-450^K^_ rev | TCT GGC CAA AAC GTC **CTT CAG TGC GTC** GAC CAG CTT CTT C |
| YL252 | LZ(3)-450^K^_ for | G AAG AAG CTG GTC **GAC GCA CTG** GAC GTT TTG GCC AGA |
| YL253 | LZ(3)-450^K^_ rev | TCT GGC CAA AAC GTC **CAG TGC GTC** GAC CAG CTT CTT C |
| YL254 | LZ(0)-450^K^_ for | G AAG AAG CTG GTC GAC GTT TTG GCC AGA |
| YL255 | LZ(0)-450^K^_ rev | TCT GGC CAA AAC GTC GAC CAG CTT CTT C |
| YL258 | C435R_for | GCC ACC GCC *CGC* CTC TGT CAC |
| YL259 | C435R_rev | GTG ACA GAG *GCG* GGC GGT GGC |
| YL260 | BamHI_450_for | GG GGA TCC GAC GTT TTG GCC AGA CGA |
| YL263 | LZ(-1)-450^K^_ for | CTG AAG AAG CTG GAC GTT TTG GCC AGA |
| YL264 | LZ(-1)-450^K^_ rev | TCT GGC CAA AAC GTC CAG CTT CTT CAG |
| YL265 | LZ(-2)-450^K^_ for | AGA CTG AAG AAG GAC GTT TTG GCC AGA |
| YL266 | LZ(-2)-450^K^_ rev | TCT GGC CAA AAC GTC CTT CTT CAG TCT |
| YL267 | LZ(-1)-438^K^_ for | CTG AAG AAG CTG CAC TAT ATC GAT GTT |
| YL268 | LZ(-1)-438^K^_ rev | AAC ATC GAT ATA GTG CAG CTT CTT CAG |
| YL269 | LZ(-2)-438^K^_ for | AGA CTG AAG AAG CAC TAT ATC GAT GTT |
| YL270 | LZ(-2)-438^K^_ rev | AAC ATC GAT ATA GTG CTT CTT CAG TCT |
| YL365 | LZ(1)-426^K^_ for | G AAG AAG CTG GTC **GAC** ATT CAG CTT CTT GAA C |
| YL366 | LZ(1)-426^K^_ rev | G TTC AAG AAG CTG AAT **GTC** GAC CAG CTT CTT C |
| YL367 | LZ(2)-426^K^_ for | G AAG AAG CTG GTC **GAC GCA** ATT CAG CTT CTT GAA C |
| YL368 | LZ(2)-426^K^_ rev | G TTC AAG AAG CTG AAT **TGC GTC** GAC CAG CTT CTT C |
| YL369 | LZ(-1)-426^K^_ for | CTG AAG AAG CTG ATT CAG CTT CTT GAA C |
| YL370 | LZ(-1)-426^K^_ rev | G TTC AAG AAG CTG AAT CAG CTT CTT CAG |
| YL371 | LZ(-2)-426^K^_ for | AGA CTG AAG AAG ATT CAG CTT CTT GAA C |
| YL372 | LZ(-2)-426^K^_ rev | G TTC AAG AAG CTG AAT CTT CTT CAG TCT |
| YL385 | virA449^+1^_for | GT AAG CAG ACC GAA TGC **GCT** GAC GTT TTG GCC AG |
| YL386 | virA449^+1^_rev | CT GGC CAA AAC GTC **AGC** GCA TTC GGT CTG CTT AC |
| YL403 | virA449^+2^_for | GT AAG CAG ACC GAA TGC **GAC GCT** GAC GTT TTG GCC AG |
| YL404 | virA449^+2^_rev | CT GGC CAA AAC GTC **AGC GTC** GCA TTC GGT CTG CTT AC |
| YL405 | virA449^+4^_for | GT AAG CAG ACC GAA TGC **GAC GCT CTG AAG** GAC GTT TTG GCC AG |
| YL406 | virA449^+4^_rev | CT GGC CAA AAC GTC **CTT CAG AGC GTC** GCA TTC GGT CTG CTT AC |
| YL411 | K298E_for | GAG CTA ATC *GAA* GAG ATC GGA GTA |
| YL412 | K298E_rev | TAC TCC GAT CTC *TTC* GAT TAG CTC |
| YL417 | virA449^+3^_for | GT AAG CAG ACC GAA TGC **GAC GCT CTG** GAC GTT TTG GCC AG |
| YL418 | virA449^+3^_rev | CT GGC CAA AAC GTC **CAG AGC GTC** GCA TTC GGT CTG CTT AC |
